# Intra-Abdominal Lipopolysaccharide Clearance and Inactivation in Peritonitis: Key Roles for Lipoproteins and the Phospholipid Transfer Protein

**DOI:** 10.3389/fimmu.2021.622935

**Published:** 2021-05-12

**Authors:** Maxime Nguyen, Gaëtan Pallot, Antoine Jalil, Annabelle Tavernier, Aloïs Dusuel, Naig Le Guern, Laurent Lagrost, Jean-Paul Pais de Barros, Hélène Choubley, Victoria Bergas, Pierre-Grégoire Guinot, David Masson, Belaid Bouhemad, Thomas Gautier

**Affiliations:** ^1^ Department of Anesthesiology and Intensive Care, Dijon University Hospital, Dijon, France; ^2^ Université Bourgogne Franche-Comté / Agrosup, Lipids Nutrition Cancer (LNC) UMR1231, Dijon, France; ^3^ INSERM, LNC UMR1231, Dijon, France; ^4^ FCS Bourgogne-Franche Comté, LipSTIC LabEx, Dijon, France; ^5^ Lipidomic Analytical Platform, Université Bourgogne Franche-Comté (UBFC), Dijon, France; ^6^ Laboratory of Clinical Chemistry, François Mitterrand University Hospital, Dijon, France

**Keywords:** endotoxemia, peritonitis, sepsis, phospholipid transfer protein, lipoproteins, inflammation, lipopolysaccharides 4

## Abstract

**Introduction:**

During peritonitis, lipopolysaccharides (LPS) cross the peritoneum and pass through the liver before reaching the central compartment. The aim of the present study was to investigate the role of lipoproteins and phospholipid transfer protein (PLTP) in the early stages of LPS detoxification.

**Material and Methods:**

Peritonitis was induced by intra-peritoneal injection of LPS in mice. We analyzed peritoneal fluid, portal and central blood. Lipoprotein fractions were obtained by ultracentrifugation and fast protein liquid chromatography. LPS concentration and activity were measured by liquid chromatography coupled with mass spectrometry and limulus amoebocyte lysate. Wild-type mice were compared to mice knocked out for PLTP.

**Results:**

In mice expressing PLTP, LPS was able to bind to HDL in the peritoneal compartment, and this was maintained in plasma from portal and central blood. A hepatic first-pass effect of HDL-bound LPS was observed in wild-type mice. LPS binding to HDL resulted in an early arrival of inactive LPS in the central blood of wild-type mice.

**Conclusion:**

PLTP promotes LPS peritoneal clearance and neutralization in a model of peritonitis. This mechanism involves the early binding of LPS to lipoproteins inside the peritoneal cavity, which promotes LPS translocation through the peritoneum and its uptake by the liver.

## Introduction

Intra-abdominal infection, which is known as peritonitis, is the second most common infection site leading to septic shock (1). Secondary peritonitis is caused by the perforation of a hollow organ (2), resulting in the release of the digestive content into the abdominal cavity. Lipopolysaccharides (LPS), also known as endotoxins, are components of the outer membrane of gram-negative bacteria. LPS, which are composed of a polysaccharide chain and a lipid A moiety, are responsible for the interaction with the innate immune system and the triggering of inflammation (3). The noxious properties of LPS are due to its interaction with the host’s immune system and its over-activation (4). Once in the blood, LPS can bind to the LPS binding protein (LBP). This complex binds to CD14 and toll-like receptor 4 (TLR4). LPS subsequently forms a complex with myeloid differentiation factor 2 (MD-2) which activates innate immunity through intra-cellular pathways (5). In parallel with the classical TLR4 pathway, intra-cellular receptors for LPS have been described (caspase-11 in mice and caspase 4/5 in humans) (6).

There are several mechanisms for LPS detoxification. First, LPS might be cleared by immune cells after monomerization of LPS aggregate by LBP. CD14 could also transfer LPS to lipoproteins. However, both of those clearance process might also enhance TLR4 related inflammation (7). One of the main pathways for LPS clearance and inactivation is the “reverse lipopolysaccharide transport” (RLT) (8). In this pathway, once in blood, LPS is rapidly transferred to lipoproteins, mainly high density lipoproteins (HDL) (9). This first step is facilitated by the phospholipid transfer protein (PLTP) which has the ability to transfer LPS to lipoproteins, especially HDL. Once bound to HDL, and resulting from masking of the lipid A moiety, LPS no longer activates TLR4. Instead, LPS is transported by lipoproteins to the liver which is the main organ involved in LPS detoxification. Indeed, both Kupffer cells and hepatocytes might inactivate LPS by enzymatic degradation catalyzed by acyloxyacyl hydrolase and alkaline phosphatase, respectively (7). Receptors involved in this process include LDL receptor and scavenger receptor class B type 1 (10–12). Thus, RLT pathway modulates inflammation and might decrease the consequences of the host response to infection by contributing to LPS neutralization and elimination. Interspecies specificities have been described between mice and human regarding response to LPS and their analogues (13). In particular, the LPS doses needed to trigger a cytokine storm are much higher in mice than in humans (14) and gene expression pattern and time course of pathways related to inflammatory response to endotoxemia significantly differ between mice and humans (15). The differences in lipoprotein profiles with LDL and HDL as main lipid carriers in humans and mice, respectively might partly explain these differences since LDL and HDL differ in their efficiency to bind LPS and in their metabolic fate (9).

PLTP is a lipid transfer protein that belongs to the lipid transfer lipopolysaccharide binding protein (LT/LBP) family. PLTP was first described for its role in phospholipid transfer between lipoprotein classes, HDL remodeling, including generation of pre-beta HDL (16) and its impact on onset and progression of atherosclerosis (17). As member of the same LT/LBP family, PLTP has structure similarities with LBP and both are able to bind LPS. In previous works, we demonstrated that PLTP played a pivotal role in the RLT pathway and overall protection against endotoxemia and sepsis ex-vivo and in animals models. Indeed, after studying a model of PLTP-deficient mice, we previously reported that PLTP reduced host susceptibility to endotoxemia (18). Furthermore, administration of recombinant human PLTP improved survival in mice following injections of LPS or bacterial infection (19). Interestingly, it has also been shown that the time to maximal blood concentration (Tmax) after intraperitoneal injection of LPS seemed longer in PLTP-deficient mice, suggesting delayed peritoneal clearance (18).

During intra-abdominal sepsis, LPS are located in the peritoneal compartment. LPS cross the peritoneum to reach the portal blood flow through the liver prior reaching the central compartment. However, little is known about the factors affecting the passage of LPS from the peritoneal cavity to portal blood through the peritoneum or about the accumulation of LPS in central blood after they pass through the liver. Furthermore, although lipoproteins are known to play a key role in LPS detoxification, data on the lipoprotein profile in peritoneal fluid are scarce, and the ability of peritoneal lipoproteins to bind LPS at the initial site of infection during peritonitis is unknown. The aim of the present study was to investigate the role of PLTP and lipoproteins in the detoxification of LPS from the peritoneal cavity, considering the 2 biological barriers (peritoneum and the liver) and the 3 compartments (peritoneal, portal and central). Our working hypothesis was that, in abdominal endotoxemia, PLTP and lipoprotein might enhance LPS clearance and neutralization by promoting the binding of LPS to lipoproteins, therefore preventing the triggering of inflammation. Our experiments were conducted at early time points in order to identify neutralizing mechanisms that occur in the very first steps in the inflammation process and that could explain the higher inflammation and mortality reported in absence of PLTP.

## Materials and Methods

### Design

The protocol was prepared before the study. Abdominal endotoxemia was modeled by intraperitoneal LPS injection in mice. LPS from *E. coli* (O55:B5 strain) was injected to mature mice (more than 6 weeks old). Mice were matched by age and sex prior to experiments. Two genotypes were used: wild-type mice (C57BL/6) (WT) and mice knocked-out for PLTP (*Pltp* ^-/-^). Every set of experiments included a control of each genotype which received intra-peritoneal (IP) injection of LPS-free phosphate buffered saline (PBS, vehicle). Every sampling procedure (except caudal sampling) was performed under inhaled anesthesia (isoflurane) titrated to maintain spontaneous breathing. The study did not have human endpoints. The manuscript is drafted in agreement with the ARRIVE 2.0 guidelines.

### Animal Procedures

LPS injection: Purified LPS of Escherichia coli serotype 055-B5 (Sigma Aldrich) was suspended in endotoxin-free PBS and vigorously mixed before use. The mice received a single injection (1mg/kg or 25mg/kg of body weight) of LPS intraperitoneally (20). For peritoneal lavage: 5ml of sodium chloride 0.9% was injected inside the peritoneal cavity. The abdomen was shaken, and the intra-abdominal fluid was collected by a second puncture. Portal sampling was performed after laparotomy on visual control under anesthesia. The time between portal and cardiac (central) sampling was less than 1 minute. After procedures, mice were sacrificed by cervical dislocation (21). Plasma was obtained by blood centrifugation (10 min, 6000*g* at 4°C). Time points for sampling were selected based on previous kinetics data (22).

### Study Approval

All animal procedures were approved by the University of Burgundy’s Ethics Committee on the Use of Laboratory Animals (registered under the number 5459). All animal procedures were performed in agreement with ethical principles.

### Lipoprotein Profile Analyses

The different fractions of plasma and of peritoneal liquid were separated according to their density by sequential ultracentrifugation in a TLA100.1 rotor (Beckman, Palo Alto, CA). Two or three ultracentrifugation steps were conducted at the appropriate densities in order to separate the plasma into 3 or 4 fractions: either 1) the triglyceride rich fraction (*d* < 1.006), the cholesterol-rich lipoprotein fraction (1.006 < *d* < 1.21) and the lipoprotein-free fraction (*d* > 1.21) or 2) the triglyceride rich fraction (*d* < 1.006), the LDL fraction (1.006 < *d* < 1.063), the HDL fraction (1.063 < *d* < 1.21) and the lipoprotein-free fraction (*d* > 1.21) were separated.

The different fractions of plasma and of peritoneal liquid were also separated by fast protein liquid chromatography (FPLC). Plasma and peritoneal liquid were pooled and 200 µl of the mix were injected in an agarose superpose 6 column and eluted in Tris/Saline/EDTA (TSE) buffer. 300µl fractions were collected.

Lipoprotein electrophoresis was performed on agarose gel (Biotec-Fisher GmbH, Reiskirchen, Germany), and manufacturer instructions were followed.

### LPS Quantitation and Activity Measurements

LPS was quantified by liquid chromatography coupled with mass spectrometry (LCMS2) (23). This method relies on the quantification of 3-hydroxymyristate (3HM) (a fatty acid from lipid A specific of LPS). Samples (*ca* 30µl) were mixed with 4µL of 3-OH-tridecanoic acid used as internal standard (1µg/µL in ethanol) and 300µL of hydrochloric acid 8M. After 3 hours of hydrolysis at 90°C, free fatty acids were extracted with 600µl of distilled water and 5ml of hexane/ethyl acetate (3/2 v/v). After evaporation of the organic phase, dried extracts were solubilized in ethanol (50µL) and 3µL were injected on a SBC18 2.1x50 mm, 1.8 µm column and connected to an Infinity 1290 HPLC system (Agilent Technologies). Separation of 3OH-free fatty acids was achieved at 45°C using ammonium formate 5mM/formic acid 0.1% as eluent A and acetonitrile 95% as eluent B. The elution gradient was set up at a flow rate of 0.4 ml/min as follows: 55% A for 0.5 min, up to 100% B in 2.5 min and maintained at 100% B for 5 min. MS/MS detection was performed in negative mode using a QqQ 6490 triple quadruple mass spectrometer equipped with a JetStream ESI source (Gas Temperature 290°C, Gas Flow 19 l/min, Nebulizer 20 psi, Sheath Gas Heater 175°C, Sheath Gas Flow 12 l/min, Capillary Voltage 2000 V, Charging 200 V). The mass spectrometer was set up in the selected reaction monitoring mode for the quantification of selected ions as follows: for 3-hydroxytetradecanoic acid (3HM), precursor ion 243.2 Da, product ion 59 Da; for 3-hydroxytridecanoic acid (IS), precursor ion 229.2 Da, product ion 59 Da, Collision energy and Cell Acceleration was set at 12 V and 2 V, respectively.

LPS activity was measured by limulus amoebocyte lysate (LAL) using a commercially available colorimetric activity assay (HycultBiotech Inc., Wayne, PA).

### Lipid Measurements

Triglycerides, cholesterol and phospholipids were measured enzymatically by colorimetry with commercially available kits (DiaSys Diagnostic Systems GmbH, Holtzheim, Germany), according to manufacturer’s instructions. Cholesterol concentrations in FPLC fractions from peritoneal fluid (**Figure 2B**) were measured by mass spectrometry, as previously described (24).

### Definitions

Portal to central plasma gradient was calculated as follows: *portal concentration – central concentration*. The hepatic first pass effect was calculated as portal to central gradient divided by portal concentration.

Cholesterol-rich lipoprotein fractions refer to HDL plus LDL fractions. Because mice are known to have only small amounts of LDL, those two density fractions were not separated in every experiment.

### Statistics

Data were collected using Microsoft Excel for Office 365. Qualitative data are presented as frequencies and percentage. Quantitative data are presented as median and interquartile range. Only non-parametric tests were carried out due to the small sample sizes. Paired data were processed using a Wilcoxon signed-rank test. The threshold for statistical significance was set to p<0.05. Statistical analysis was performed with R Studio Version 1.2.5001^©^ 2009-2019 RStudio, Inc. Graphical representations were made using the “ggplot2” package. The experimental unit was one animal. Sample sizes were chosen as a compromise between power and the three Rs principle. All collected data were analyzed. Only 4 mice without LPS in the peritoneal fluids and blood after intraperitoneal LPS injection were excluded from the analysis (considered as injection error).

## Results

### Lipoprotein Profile in Peritoneal Fluid, Portal Blood and Peripheral Blood at Basal State

**Figure 1:**
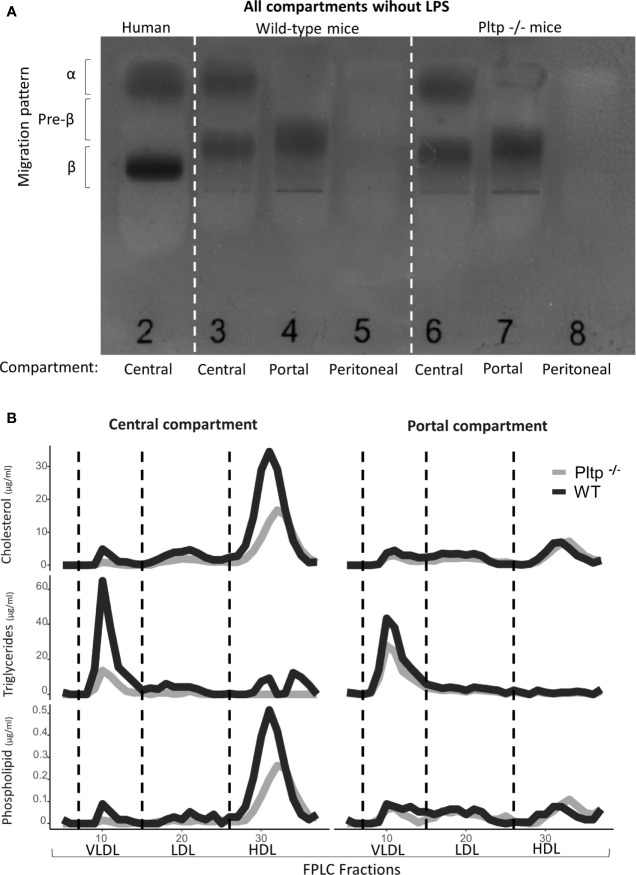
Characterization of lipoproteins in central, portal, and peritoneal compartments (from pooled samples) in wild-type (black lines) and *Pltp* ^-/-^ (grey lines) mice in absence of LPS injections by **(A)** Electrophoresis and **(B)** Fast protein liquid chromatography. Both electrophoresis and FPLC showed clear differences in lipoprotein composition between portal and central blood. For FPLC, fractions 5 to 37 are shown. FPLC, Fast protein liquid chromatography; Pltp -/-, Knock out for the phospholipid transfer protein; VLDL, very low density lipoprotein; LDL, low density lipoprotein; HDL, High density lipoprotein.

Peritoneal fluid, portal plasma and central plasma were sampled from 8 wild-type (WT) and 8 PLTP knock-out (*Pltp* ^-/-^) mice in the absence of LPS injection. Fast protein liquid chromatography (FPLC) analysis was conducted on pooled samples for each compartment (central plasma, portal plasma and peritoneal fluid) and lipoprotein subpopulations were analyzed by electrophoresis. Results are shown in **Figure 1**. In the peritoneal fluid, small amounts of lipoproteins (in the pre-beta and beta migration range) were detected by agarose gel electrophoresis (**Figure 1A**). However, because the peritoneal lavage resulted in dilution, lipid characterization could not be conducted on FPLC fractions. Comparing the central and portal compartments, HDL concentration appeared clearly higher in the former compartment, with consistent observations using either FPLC or electrophoresis and with similar profiles whether PLTP was expressed or not (**Figure 1**).

### PLTP Promotes Peritoneal LPS Clearance and Inactivation

**Figure 2:**
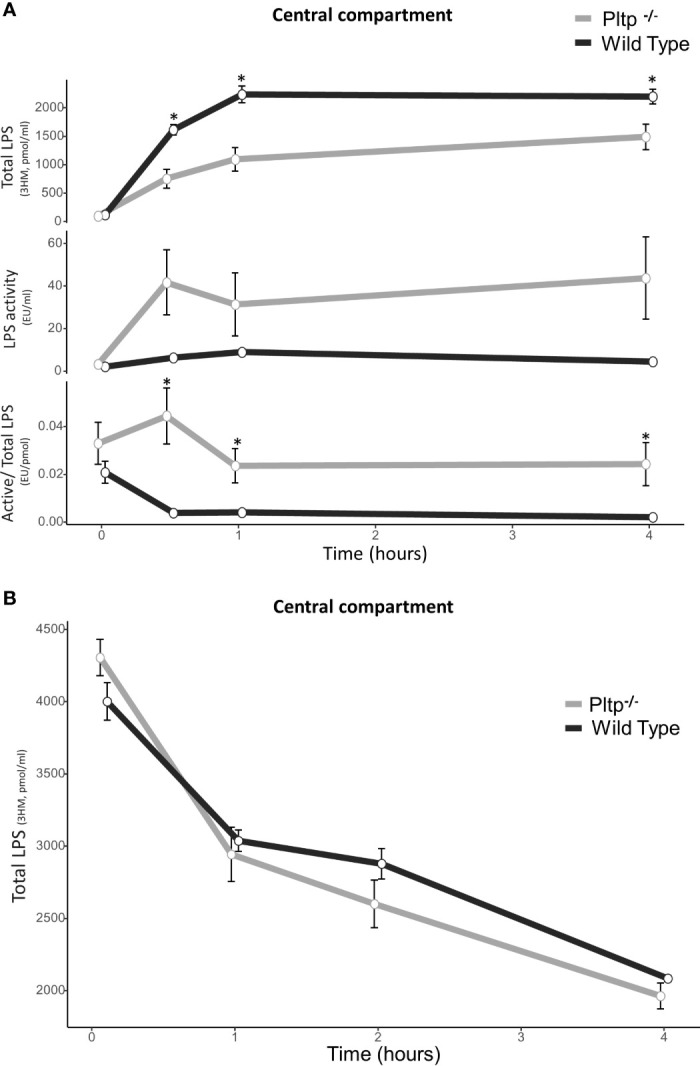
Kinetics of central LPS concentration and activity in wild-type (black lines) and *Pltp* ^-/-^ (grey lines) mice. **(A)** After intra-peritoneal injection (n = 8 vs. n = 7) and **(B)** after caudal (intravenous) injection (n = 8 vs. n = 8). **(A)** After intraperitoneal injection, wild type mice had higher LPS absorption, but absorbed LPS was less active. **(B)** After intravenous injection, there was no differences in LPS concentrations. LPS, lipopolysaccharide; 3HM, 3-hydroxymyristate; WT, wild-type; Pltp -/-, Knock out for the phospholipid transfer protein. Results are presented as means +/- SEM; *, statistically significant difference (p<0.05).

After intraperitoneal injection of LPS (1mg/kg) in WT and *Pltp* ^-/-^ mice (n=9 in each group), the total concentration and biological activity of LPS were measured in central blood using the liquid chromatography coupled with mass spectrometry (LCMS2) test and limulus amoebocyte lysate (LAL) test, respectively (**Figure 2A**). In the presence of PLTP, LPS molecules appeared more quickly in the central blood, and higher concentrations were detected at 30 minutes, 1 hour, and 4 hours. However, LPS biological activity as assessed by the LAL test tended to be lower as compared to *Pltp* ^-/-^ mice, and specific LPS activity was significantly reduced in WT mice (p<0.05) (**Figure 2A**). **Figure 2B** shows LPS kinetics following intravenous injection of a single, 1mg/kg LPS dose. At the early time points studied, plasma decay of LPS occurred at similar rates in *Pltp* ^-/-^ (n=7) and WT mice (n=8).

### PLTP Facilitates LPS Peritoneal Translocation

**Figure 3:**
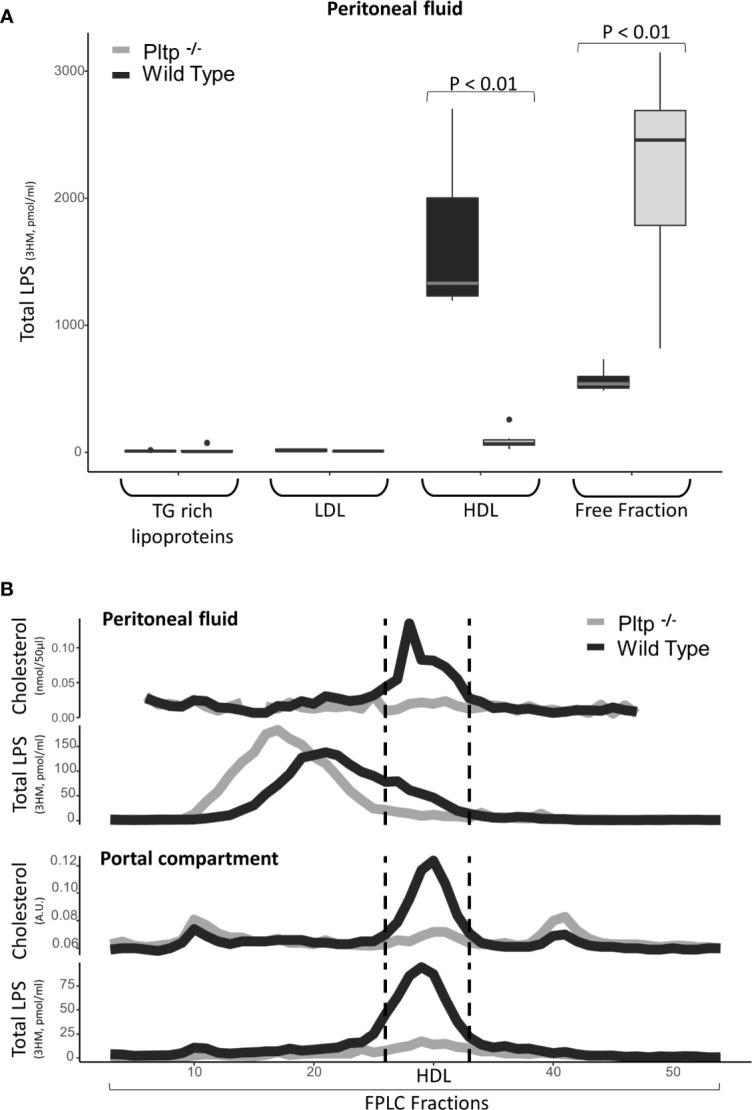
Characterization of LPS distribution in lipoprotein fractions in portal and peritoneal compartments 30 minutes after LPS injection in wild-type (black) and *Pltp* ^-/-^ (grey) mice. **(A)** LPS was quantified in lipoprotein fractions individualized by ultracentrifugation (n=7 in both groups) in peritoneal fluids. **(B)** LPS and cholesterol were quantified in lipoprotein fractions individualized by FPLC (from pooled samples) in both peritoneal fluid and portal blood. Both methods support an association between lipoproteins and LPS in the peritoneal cavity and in portal blood, but only in wild-type mice. *Pltp* ^-/-^ mice appeared to have lower HDL levels. LPS, lipopolysaccharide; FPLC, Fast protein liquid chromatography; 3HM, 3-hydroxymyristate; WT, Wild type; Pltp -/-, Knock out for the phospholipid transfer protein; TG, Triglycerides; LDL, Low density lipoproteins; HDL, High density lipoproteins. Total LPS was measured by mass spectrometry. Total LPS and cholesterol are expressed as arbitrary (A.U.). Box represents median and interquartile range, bar represents minimal and maximal range, points are outliers. P-values are given only if p < 0.05.

**Figure 4:**
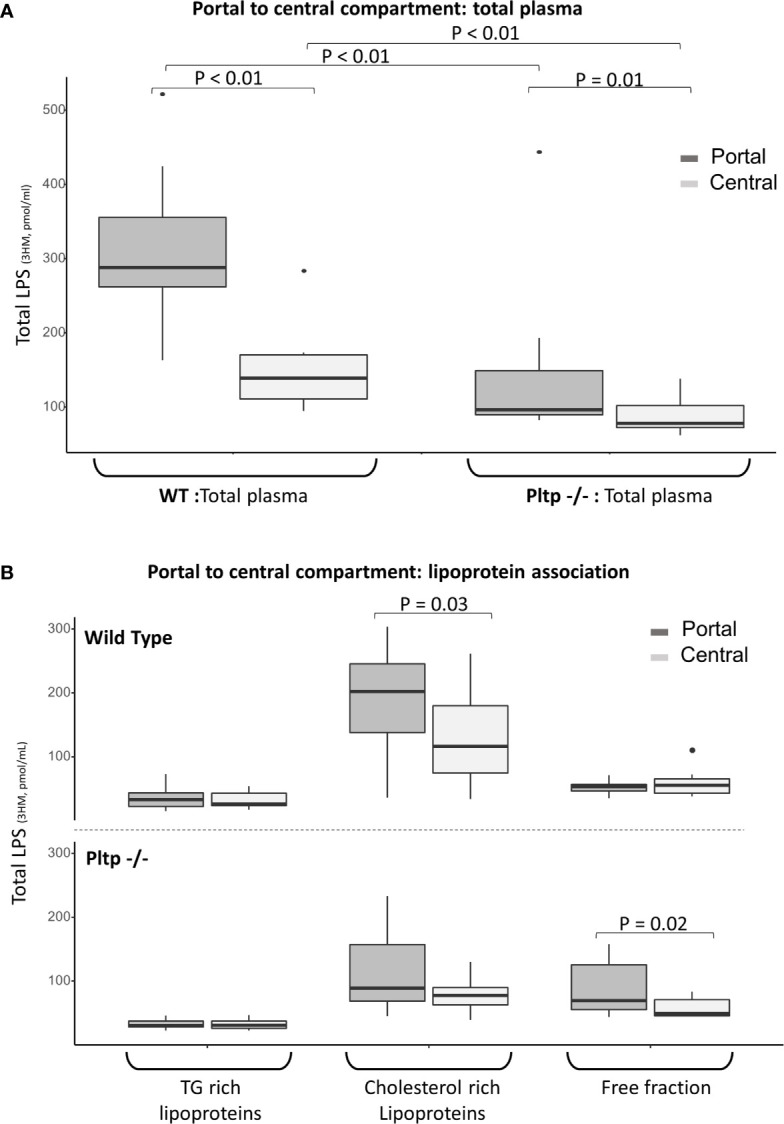
Hepatic first pass effect 15 minutes after 1mg/kg LPS injection in wild-type and *Pltp* ^-/-^ mice. **(A)** LPS was quantified in the portal (dark grey) and central (light grey) compartments in total plasma of WT and *Pltp* ^-/-^ mice (n=10 vs. n= 10). **(B)** LPS was quantified in lipoprotein fractions individualized by ultracentrifugation in blood from portal (dark grey) and central (light grey) compartment in WT and *Pltp* -/- mice (n=12 vs. n= 10). Both genotypes exhibited a significant hepatic first pass effect. This effect was greater in wild-type mice, in which the difference in LPS concentration was located within the lipoprotein fraction, whereas it was located within the free fraction in *Pltp* ^-/-^ mice. LPS, lipopolysaccharide; 3HM, 3-hydroxymyristate; WT, wild-type; Pltp -/-, Knock out for the phospholipid transfer protein; TG, triglycerides. Boxes represent median and interquartile range, bars represent minimal and maximal range, dots are outliers. P-values are given only if p < 0.05.

Peritonitis was modeled by intra-peritoneal LPS injection (1mg/kg). Nine *Pltp* ^-/-^ mice were compared to eight WT mice. Thirty minutes after the LPS injection, peritoneal fluid and portal blood were drawn and components in peritoneal lavage were fractionated by FPLC gel permeation chromatography and ultra-centrifugation. The results are presented in **Figure 3**. In peritoneal fluid, LPS was located in lipoprotein-containing fractions, mainly HDL, in higher proportion in WT mice than in *Pltp* ^-/-^ mice (59% [49;67] vs. 9% [7;13], p <0.01) (**Figure 3A**). FPLC analysis revealed that LPS co-eluted mostly with cholesterol in WT mice but not in *Pltp* ^-/-^ mice, suggesting an association of LPS with lipoproteins when PLTP is expressed. Under inflammatory conditions, it appeared that HDL cholesterol levels were higher in peritoneal fluid and portal blood in WT mice than in *Pltp* ^-/-^ mice (**Figure 3B**). In portal blood, LPS appeared in higher concentrations in WT mice (**Figures 3B** and **4A**). LPS was mainly located within the HDL fraction when separated by FPLC (**Figure 3B**).

### PLTP Promotes a Hepatic First Pass Effect on LPS

Fifteen minutes after the intraperitoneal LPS injection (1 mg/kg), portal and central (cardiac) blood were drawn from WT and *Pltp* ^-/-^ mice (n=10 in each group). Overall, LPS concentration was higher in WT mice than in *Pltp* ^-/-^ mice, both in the portal (288 [262-355] vs. 97 [90-149] pmol/ml, p < 0.01) and the central compartments (139 [111-170] vs. 78 [72-102] pmol/ml, p<0.01), respectively (**Figure 4A**). Significant portal to central gradients were observed in both genotypes, but the hepatic first pass effect on LPS was higher in WT mice than in *Pltp* ^-/-^ mice (51% [40-61] vs. 17% [11-24], p=0.03).

In order to gain more insight into the differences in LPS lipoprotein distribution upstream and downstream of the liver, 12 WT mice were compared to 10 *Pltp* ^-/-^ mice. Portal and central plasma fractions were separated by ultracentrifugation. LPS was mostly located in the lipoprotein fractions of WT mice in both the portal (66% [62;72] for WT vs. 50% [41;54] for *Pltp* ^-/-^, p=0.01) and central compartments (61% [46;67] vs. 48% [44;51], p=0.10). The portal to central gradients of LPS for the different fractions are presented in **Figure 4B**. WT mice exhibited a significant portal to central LPS gradient in the lipoprotein fraction whereas the portal to central gradient was significant only in the lipoprotein-free fraction of *Pltp* ^-/-^ mice.

**Figure 5:**
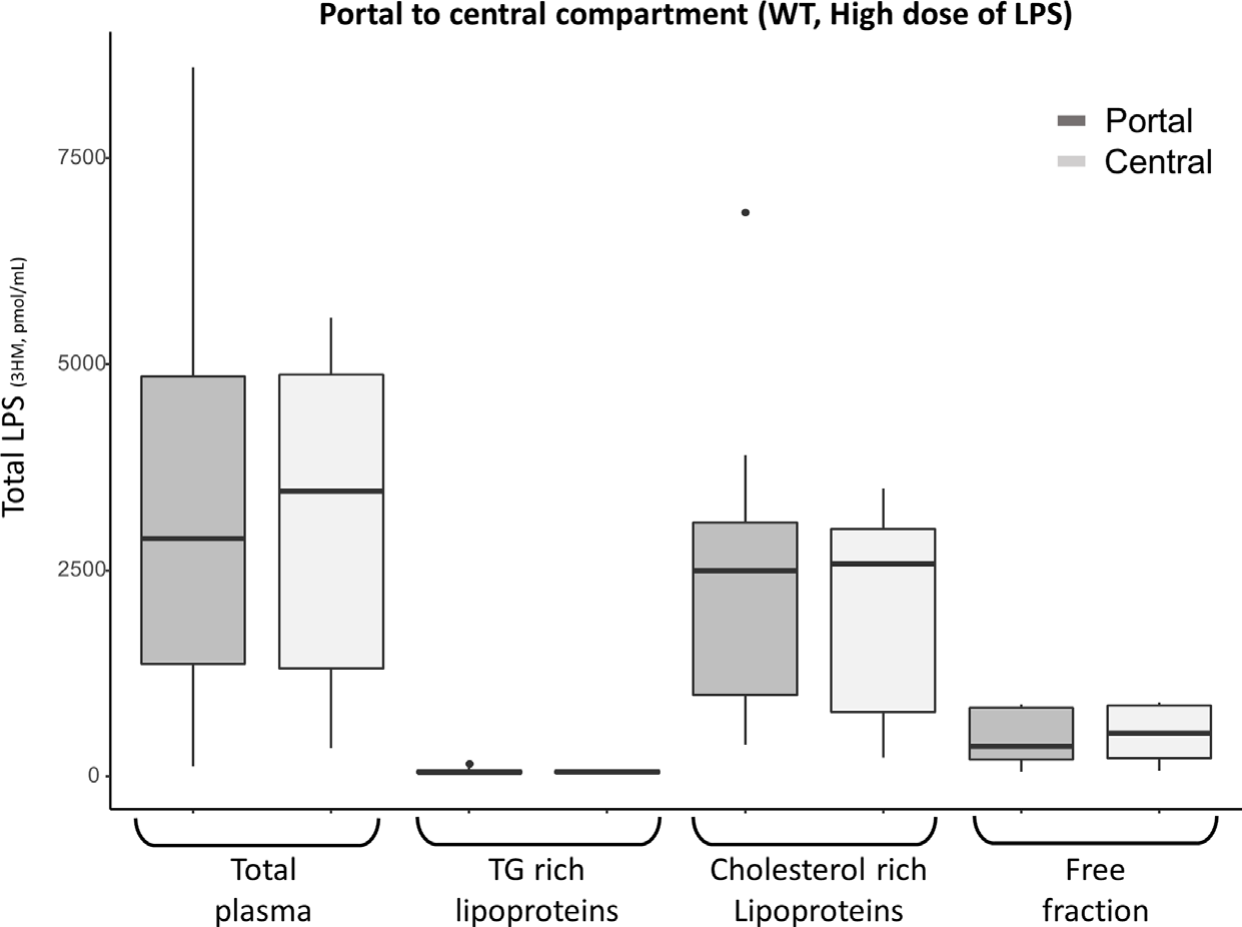
Portal (dark grey) and central (light grey) LPS concentrations 15 minutes after high dose (25 mg/kg) LPS injection in 9 WT mice. There was no portal to central LPS gradient for high LPS load, suggesting saturation of the hepatic first pass effect. After the high dose of LPS, most portal and central LPS remained located within the lipoprotein fraction in WT mice. LPS, lipopolysaccharide; 3HM, 3-hydroxymyristate; WT, wild-type; TG, Triglycerides Boxes represent median and interquartile range, bars represent minimal and maximal range, dots are outliers. All p-values are > 0.05.

In a third set of experiments, after injection of a single high dose of LPS (25 mg/kg) to 9 WT mice, the bulk of LPS was still found to be bound to the lipoprotein fraction at 15 minutes: 80% [80-90] in portal blood and 77% [74;79] in the central compartment. However, the first pass effect was no longer observed (**Figure 5**).

### LPS Associates With Human Lipoproteins Within the Peritoneal Cavity of Pltp^-/-^ Mice

**Figure 6:**
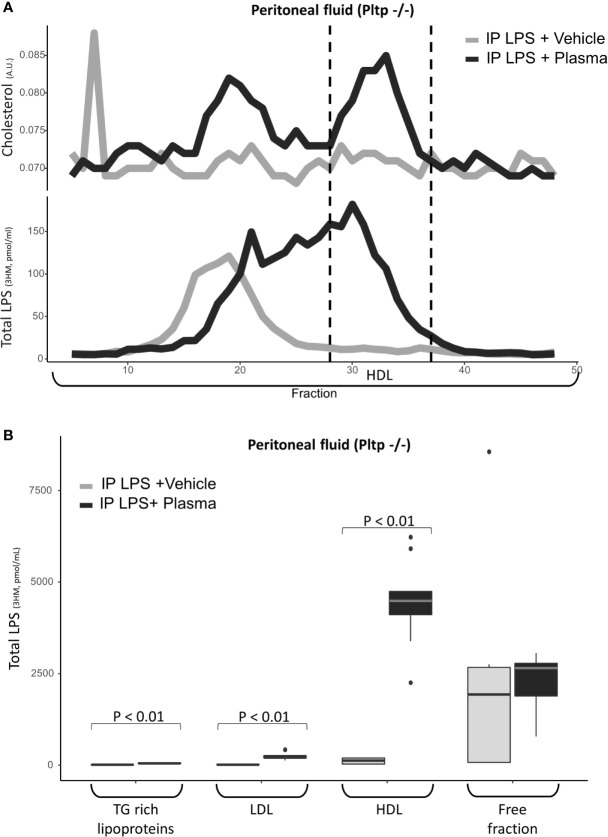
Characterization of LPS distribution in lipoprotein fractions in the peritoneal compartment 30 minutes after intraperitoneal LPS injection with co-injection of human plasma (black line) or vehicle (grey line) (n=9 vs. n=9) in *Pltp* ^-/-^ mice. **(A)** LPS and cholesterol were quantified in lipoprotein fractions individualized by FPLC (in pooled samples). **(B)** LPS was quantified in lipoprotein fractions individualized by ultracentrifugation. Both methods supported an association between LPS and lipoprotein in the peritoneum after plasma injection. LPS, lipopolysaccharide; FPLC, Fast protein liquid chromatography; 3HM, 3-hydroxymyristate; IP, intra-peritoneal; Pltp -/-, Knock out for the phospholipid transfer protein; TG, triglycerides; LDL, low density lipoproteins; HDL, high density lipoproteins. Boxes represent median and interquartile range, bars represents minimal and maximal range, dots are outliers. P-values are given only if p < 0.05.

Human plasma (7.5 µl/g) or vehicle was injected immediately after LPS in the peritoneum of *Pltp* ^-/-^ mice (n=9 per group). When the group that received the plasma injection was compared to the control group injected with the vehicle, the peritoneal fluids of the plasma injection group contained more lipoproteins, as assessed by cholesterol-containing FPLC fractions. LPS was found to associate readily with human HDL in the peritoneal fluid in the *Pltp* ^-/-^ mice treated with intra-peritoneal plasma injection (**Figures 6A, B**).

## Discussion

Our main finding is that, in a model of peritonitis, PLTP promotes the peritoneal clearance and neutralization of LPS, concurrently with binding of LPS to lipoproteins. This clearance process is initiated very early, when the binding of LPS to lipoproteins can still occur inside the peritoneal cavity. In the presence of PLTP, LPS were able to bind to lipoproteins in all three compartments (peritoneal, portal and central). PLTP-dependent binding was found to facilitate the passage of LPS through the peritoneal membrane and subsequent uptake by the liver.

In the present study, a PLTP-mediated association between LPS and lipoproteins within the peritoneal cavity is reported for the first time. This finding is in line with the ability of PLTP to transfer LPS to lipoproteins in blood (25), indicating that PLTP can act in the same way in blood plasma and peritoneal fluid. Because LPS is neutralized once it is associated with lipoproteins (8), our study suggests that LPS might be inactivated very quickly in the peritoneal cavity. Differences exists between mice and human regarding lipoprotein involved in this pathway. Mostly, mice have low level of circulating low density lipoproteins (LDL) and very low density lipoproteins (26).

After intraperitoneal administration, drugs are mostly absorbed *via* the visceral peritoneum (27). Blood from the visceral peritoneum then flows into the portal vein and through the liver before reaching the central circulation. In mice expressing PLTP, we observed a continuum in the association of LPS with lipoproteins between peritoneal fluid and portal blood. This continuum suggests that lipoproteins might facilitate the peritoneal translocation of LPS, thus explaining the higher LPS clearance observed in the presence of PLTP. Previous reports have suggested that lipoproteins and PLTP might cross the peritoneum (19, 28). Peritoneal inflammation is known to increase peritoneal permeability (29), and the increase in permeability might facilitate lipoprotein exchange between the portal and peritoneal compartment in the context of peritonitis. On the contrary, sepsis is known to lower HDL concentration (30). According to our results, one might hypothesize that low HDL concentration might impair intraperitoneal LPS clearance.

Experimental data has demonstrated lower LPS central concentration when injected into a portal vein compared to a peripheral vein, supporting the hypothesis of a hepatic first pass effect (31). The portal to central plasma LPS gradient had already been observed in the setting of digestive LPS translocation in rats (32). The gradient of LPS between portal and central blood as observed in the present study brings direct support in favor of a first pass of LPS a short time after LPS infusion.

In line with previous studies, these observations indicate that LPS readily associates with lipoproteins in a neutralizing process mediated by PLTP. Indeed, we observed higher LPS neutralization in WT mice, which can be paralleled with higher LPS binding to lipoproteins in the peritoneal and portal compartments. Indeed, the increased capacity of PLTP to neutralize LPS might explain the protective effect previously reported in sepsis (18, 19). Our results add significant knowledge regarding the key roles of lipoproteins and PLTP in LPS clearance and inactivation in abdominal sepsis. Enhancing this pathway by increasing lipoprotein levels and functionality, *in situ*, might help to increase LPS inactivation and clearance very early at the source of the insult, and protect against the harmful consequences of sepsis in patients with endotoxemia of abdominal origin.

Some limitations should be underlined. First, intraperitoneal LPS injection does not reflect the clinical setting (*i.e.* polymicrobial human peritonitis). However, the present study aimed to specifically describe mechanisms involved in lipopolysaccharide clearance and inactivation at early time points. Intraperitoneal injection allows to standardize both doses and timing, thus to increase reproducibility. In addition, this model of peritonitis enabled us to specifically study the LPS pathway by avoiding exposure to multiple pathogens associated molecular patterns, thus to precisely monitor LPS across the different compartments. Because bacteremia is a much more complex condition during which endotoxemia is only one among several burdens, infection data might have given different results. We did not study the lymphatic route. Lymphatics are also involved in the clearance of the peritoneal cavity (33). Substances absorbed by lymphatics circulate through the thoracic duct to the subclavian vein, thus bypassing the liver and the hepatic first pass. However, it is very unlikely that LPS might have reached central circulation through lymphatic route 15 or 30 minutes after intraperitoneal injection due to its slow flow (34). Intraperitoneal lipid quantification was limited because of the dilution that inevitably occurred during peritoneal lavage. However, cholesterol could be quantified by mass spectrometry on one FPLC series. We chose to study short endpoint in order to identify pathological mechanisms that occurs early in the inflammation process. Longer endpoints in particular reporting higher inflammation and mortality in *Pltp* -/- mice have not been re-explored here but have previously been described (18, 19). While it has been reported that PLTP, rather than HDL pool size was critical to reduce LPS induced inflammation (35), our model did not allow us to address this question because both PLTP and HDL metabolism are altered in *Pltp* ^-/-^ mice. We did not study the cellular compartment after peritoneal lavage. Exploring the profile of cell populations over time might have given further information about peritoneal inflammation upon LPS stimulation. For instance, it has been reported that natural killer cells that might contribute to a hyper response in sepsis by acquiring memory-like properties (36). However, *ex-vivo* data already exist and showed that peritoneal macrophage activation by LPS depended on both PLTP secretion by the cells and the presence of lipoproteins in the medium (37).

In conclusion, using a model of peritonitis we demonstrated that PLTP promotes the binding of LPS to lipoproteins, making it a key factor of peritoneal clearance and neutralization. First, this phenomenon involves the rapid and efficient PLTP-dependent binding of LPS to lipoproteins, promoting its transfer from the peritoneal cavity to the portal blood. Secondly, a hepatic first pass effect occurs, thus lowering the central LPS concentration in WT mice. This results in higher LPS clearance and inactivation when originating from the peritoneal cavity. Finally, LPS binds readily to human lipoproteins when human plasma is infused inside the peritoneal cavity of *Pltp* ^-/-^ mice. Overall, this work highlights the key role of PLTP and lipoproteins during the early steps of the absorption and clearance of LPS originating from the peritoneal cavity.

## Data Availability Statement

The raw data supporting the conclusions of this article will be made available by the authors, without undue reservation.

## Ethics Statement

The animal study was reviewed and approved by University of Burgundy’s Ethics Committee on the Use of Laboratory Animals (registered under the number 5459). Written informed consent for participation was not obtained from the owners because this was not applicable.

## Author Contributions

MN, TG, LL, BB, and DM conceived and guided the project. MN, GP, AT, NL-G, AJ, AD, J-PP, VB, HC, and TG carried out the experiments. MN, TG, and BB analyzed the data. MN, TG, and BB wrote the manuscript. TG, LL, P-GG, BB, and DM reviewed and edited the manuscript. All authors contributed to the article and approved the submitted version.

## Funding

This work was supported by a French Government grant managed by the French National Research Agency (Agence nationale de la recherche) as part of the “Investissements d’Avenir” program, reference: ANR-11-LABX-0021-01- LipSTIC Labex.

It is also part of two integrated projects funded by the European Union (FEDER) and the French government (Regional Council of Bourgogne-Franche-Comté).

## Conflict of Interest

The authors declare that the research was conducted in the absence of any commercial or financial relationships that could be construed as a potential conflict of interest.

## Abbreviations

3HM, 3-hydroxymyristate; FPLC, Fast protein liquid chromatography; HDL, High density lipoprotein; LAL, Limulus amoebocyte lysate; LBP, Lipopolysaccharide binding protein; LCMS2, Liquid chromatography coupled with mass spectrometry; LPS, Lipopolysaccharide; PBS, Phosphate buffered saline; PLTP, Phospholipid transfer protein; PLTP -/-, Knock out for PLTP; RLT, Reverse lipopolysaccharide transport; TLR4, Toll-like receptor 4; TSE, Tris/Saline/EDTA; WT, Wild type.
